# Supplementary material for: Identification of photoperiod-induced specific miRNAs in the adrenal glands of Sunite sheep (Ovis aries)
Source: Front Vet Sci. 2022 Jul 22;9:888207. doi: 10.3389/fvets.2022.888207 (PMC9354845; doi:10.3389/fvets.2022.888207)
Supplement: Supplementary file 1 [file Data_Sheet_1.ZIP › Supplementary file/miRDeep2 file.zip!/miRDeep2 file/Novel_Structure/SPLP42A3/Novel_621.pdf]

Provisional ID	:	NC_019471.2_25304
Score total	:	88.8
Score for star read(s)	:	3.9
Score for read counts	:	80.7
Score for mfe	:	2.6
Score for randfold	:	1.6
Score for cons. seed	:	
Total read count	:	170
Mature read count	:	151
Loop read count	:	0
Star read count	:	19

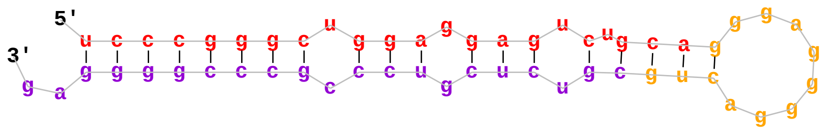

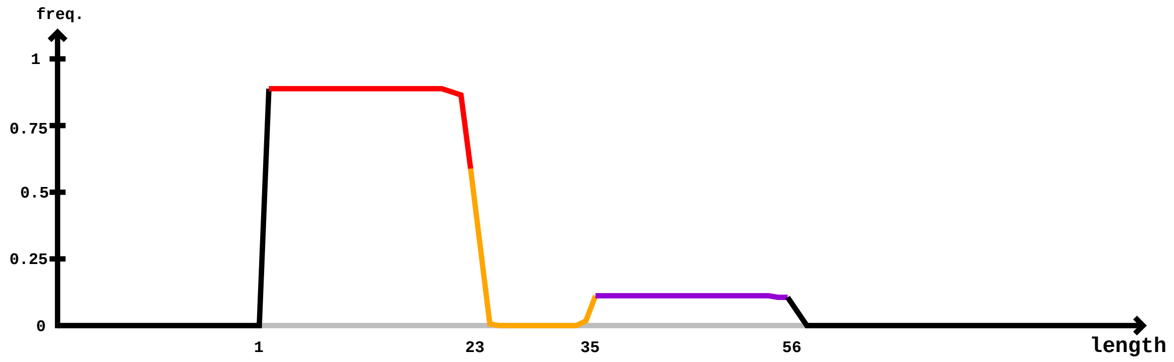

Mature **Star**

```
5'- ggaccugggcaggggcggggucccgggcuggaggagucugcagggaggggacugcgucucgucccgcccggggagaccgccccuugccgcucccaggacgguggauagcgg  -3'    obs
    ggaccugggcaggggcggggucccgggcuggaggagucugcagggaggggacugcgucucgucccgcccggggagaccgccccuugccgcucccaggacgguggauagcgg          exp
    .......(((((((((((..((((((((.(((.(((.(.((((........))))).))).))).))))))))...))))))).))))(((.(((......)))..)))..   reads       mm         sample
    ....................ucccgggcuggaggagucu........................................................................   2           0          seq
    ....................ucccgggcuggaggagucug.......................................................................   2           0          seq
    ....................ucccgggcuggaggagucugc......................................................................   47          0          seq
    ....................ucccgggcuggaggagucugca.....................................................................   67          0          seq
    ....................ucccgggcuggaggagucugcag....................................................................   32          0          seq
    ....................ucccgggcuggaggagucugcagg...................................................................   1           0          seq
    .....................................................gcgucucgucccgcccggggag....................................   2           0          seq
    .....................................................gcgucucgucccgcccggggaga...................................   1           0          seq
    ......................................................cgucucgucccgcccgggg......................................   1           0          seq
    ......................................................cgucucgucccgcccggggag....................................   12          0          seq
    ......................................................cgucucgucccgcccggggaga...................................   3           0          seq
```
